# A Summer Educational Program for Early Medical Student Exposure to Multidisciplinary Oncology: A Pilot Experience

**DOI:** 10.7759/cureus.115665

**Published:** 2026-09-02

**Authors:** Melinda Gevorgian, Leslie Harrell, Gary D Lewis, Santanu Samanta

**Affiliations:** 1 Internal Medicine, Alabama College of Osteopathic Medicine, Dothan, USA; 2 Radiation Oncology, University of Arkansas for Medical Sciences, Little Rock, USA

**Keywords:** career development, medical education, medical education and training, medical students, mentorship, oncology exposure, preclinical medical students, radiation oncology, radiation oncology education, undergraduate medical education

## Abstract

Cancer remains the second leading cause of death in the United States, highlighting the need to strengthen the oncology workforce. Undergraduate medical education often lacks sufficient exposure to oncology, particularly radiation oncology, resulting in limited awareness of the specialty. Structured rotations in oncology, along with mentorship and research opportunities, have been shown to increase interest in oncology and support entry into these fields. To address this gap, we implemented a summer rotation between the first and second years of medical school at our institution to provide early exposure to oncology. The rotation incorporated clinical experiences across multiple oncologic specialties, anchored in radiation oncology, along with research opportunities and mentorship. Two students participated in the rotation. Both rated the experience as “excellent” and subsequently contributed to scholarly work in radiation oncology, including an abstract and a manuscript on spatially fractionated radiation therapy (GRID) for the management of bulky tumors. Following medical school, one student matched into Family Medicine, while the other matched into Internal Medicine, with continued interest in pursuing a medical oncology fellowship. These findings suggest that early, structured exposure to oncology may represent an important first step in increasing medical student interest in radiation oncology and oncology-related specialties. Such multidisciplinary approaches may also support improved understanding of oncology care and facilitate early academic engagement within the field.

## Introduction

Cancer remains the second leading cause of death in the United States, underscoring the need to develop and sustain a robust oncology workforce equipped to meet current and future demands [1]. Cancer care is inherently multidisciplinary, encompassing surgery, systemic therapies such as chemotherapy and immunotherapy, and radiation therapy.

Despite the increasing burden of cancer, medical student exposure to and interest in some oncology specialties remain low, with studies reporting concerning declines in interest or applications in certain oncology disciplines [2,3].

This widespread misunderstanding of the specialty likely reflects broader systemic gaps in medical education. Radiation oncology faculty have reported that a minority of departments are involved in formal undergraduate medical education [2]. Additionally, there is limited medical student involvement in radiation oncology scholarship, with only a small proportion of authors in American Society for Radiation Oncology journals being medical students (105/1785) [4]. Furthermore, this lack of exposure is reflected in persistent knowledge gaps among trainees and physicians, including medical students and primary care providers. These gaps include difficulty identifying appropriate indications for radiation therapy, understanding which malignancies are treated with radiation, and recognizing associated toxicities. Such deficiencies may contribute to delays in appropriate referral, particularly in time-sensitive scenarios such as radiation oncology emergencies, including spinal cord compression [5].

More recently, concerns have emerged regarding declining interest in radiation oncology. Amini et al. detailed a sustained decrease in interest beginning around 2017, accompanied by an increasing number of unfilled residency positions each year. Several factors have been proposed, including concerns about future job opportunities, changes in treatment paradigms, and limited exposure to radiation oncology during medical school training [3].

Prior studies suggest that early exposure, mentorship, and structured educational experiences in radiation oncology may support student interest in and understanding of the specialty [6]. In an effort to address this well-established problem in our state, we created and implemented a structured preclinical rotation anchored in radiation oncology, with integrated exposure to multiple oncology subspecialties. The goal was to provide early, multidisciplinary insight into cancer care while highlighting the role of radiation oncology within the broader oncologic landscape.

The summer oncology educational program described in this report was developed and implemented at the University of Arkansas for Medical Sciences (UAMS) in 2022, during which all faculty co-authors were UAMS employees. The manuscript represents a collaborative scholarly effort involving program leadership, data acquisition, and manuscript preparation by the listed authors.

## Technical report

Materials and methods

We developed a preclinical medical student rotation in oncology designed to be completed during the summer between the first and second years of medical school. The rotation experience was planned to be tailored to specific student interests, and there was an expectation for students to contribute to a research project. At the conclusion of the rotation, participants completed a paper-based questionnaire assessing their perceptions of the educational experience (Appendices). The questionnaire consisted of eight closed-response items addressing staff engagement, overall experience, rotation length, and time allocation across clinical, multidisciplinary, and research experiences. Responses were selected from predefined categorical response options and summarized descriptively. Two free-response questions asked participants to identify opportunities for program improvement and the most positive aspects of the rotation; these responses were reviewed descriptively. No pre-rotation survey or formal baseline assessment of oncology interest was conducted; therefore, the questionnaire was not used to assess changes in oncology interest, knowledge, or awareness.

A call for applications to first-year medical students was distributed in spring 2022. Strong consideration was given to women or under-represented minorities, although this was not a requirement. Students were offered a stipend of $3,500, of which $500 was designated to support publication and presentation costs related to their research projects. Funding for the program was provided by the Arkansas Medical Society through the Medical Education Foundation for Arkansas grant mechanism, which supports educational initiatives, including the development of structured preclinical experiences such as this oncology rotation.

Institutional Review Board approval was not required because no patient- or student-identifying information was collected. Questionnaire responses were reported without identifying participant information.

Program structure

The seven-week rotation was conducted from June 6 through July 29, 2022, at the University of Arkansas for Medical Sciences (UAMS). The program began with an introductory overview of oncology and the goals of the rotation. Students were exposed to multiple oncology-related disciplines through clinical shadowing experiences, multidisciplinary clinical exposure, mentorship, and participation in research activities. Clinical experiences included exposure to radiation oncology, medical oncology, colorectal surgery, cardiothoracic surgery, urologic oncology, and sarcoma surgery clinics. Students observed patient consultations, treatment-planning discussions, and multidisciplinary decision-making.

Radiation oncology served as the anchor specialty of the rotation. Students also participated in regularly scheduled educational activities within the Radiation Oncology Department, including faculty-led and resident-led didactics, radiation biology and radiation physics didactics, chart rounds, and multidisciplinary tumor boards.

Students participated in departmental radiation oncology research activities, including exposure to basic science laboratory research and contribution to scholarly projects. The program concluded with a final presentation in which participants summarized key concepts learned during the rotation and reflected on their experiences within multidisciplinary oncology care.

Results

Two students entering their second year of medical school at the UAMS (Class of 2025) applied to the rotation, and both were accepted following departmental review. Day-to-day operations were coordinated by a radiation oncologist, program coordinator, and senior medical student, who also served as points of contact for participants and faculty.

Both participants rated the overall rotation experience as “excellent.” Both reported that the rotation duration, time spent in the radiation oncology clinic, time shadowing radiation oncology attending physicians and other cancer physicians, and time spent in radiation oncology laboratory research were “about right.” Both participants indicated that they would have preferred more time with radiation therapists, dosimetrists, and physicists. Both also indicated that staff were knowledgeable and eager to support their participation.

In free-response feedback, participants highlighted their appreciation for the clinical and academic learning experiences and specific multidisciplinary experiences, including treatment planning and patient interaction. Suggested improvements were primarily logistical, including more consistent communication regarding weekly schedules and shadowing opportunities. Participants also indicated a desire for greater exposure to radiation therapists, dosimetrists, and physicists.

Both participants subsequently contributed to scholarly work in radiation oncology, resulting in a published conference abstract and subsequent peer-reviewed publication focused on spatially fractionated radiation therapy (GRID) for bulky tumors. One participant subsequently entered Family Medicine and the other Internal Medicine; the latter has expressed interest in pursuing fellowship training in medical oncology.

## Discussion

Our structured multidisciplinary rotation provided two preclinical medical students with early oncology exposure anchored in radiation oncology. Both participants rated the overall rotation experience as “excellent” and subsequently engaged in radiation oncology scholarship. Given the small cohort and absence of a baseline assessment or a comparison group, these findings should be interpreted as a descriptive pilot experience rather than evidence of an effect on oncology interest or specialty choice.

Prior studies provide context for this experience. Structured radiation oncology educational programs have reported improvements in participants’ understanding of the specialty and, when measured prospectively, interest in radiation oncology [7,8]. Longer-term mentorship experiences have also been associated with substantial scholarly activity and subsequent entry into radiation oncology among some participants [6]. Unlike these studies, however, our program did not include a pre-rotation assessment or a formal measure of career interest. Accordingly, our findings should be interpreted as descriptive participant experiences rather than evidence of a change attributable to the rotation.

Both participants indicated that they would have preferred additional time with radiation therapists, dosimetrists, or physicists. This finding may reflect aspects of the rotation itself but may also highlight broader challenges in fully integrating multidisciplinary team members into early educational experiences. In this context, our rotation was intentionally designed to highlight radiation oncology within a multidisciplinary framework, providing exposure to its role alongside surgical and medical oncology. Participants reported consistently positive experiences, with both rating the rotation as “excellent” and emphasizing the clinical and academic value of the experience. In addition to subjective satisfaction, both participants contributed to scholarly work in radiation oncology, resulting in a published conference abstract and subsequent peer-reviewed publication focused on the application of spatially fractionated radiation therapy (GRID) for bulky tumors.

Neither participant subsequently entered radiation oncology. One entered Family Medicine and the other Internal Medicine; the latter has subsequently expressed interest in pursuing fellowship training in medical oncology. These career outcomes are descriptive and cannot be attributed to participation in the rotation.

Importantly, this pilot experience had several key limitations. It represents a single-institution experience with a very small, self-selected cohort (n = 2), limiting generalizability and introducing substantial potential for selection bias. No baseline assessment or comparison group was included, and no objective assessment of knowledge, competency, or other educational outcomes was performed. The post-rotation questionnaire was used for program evaluation and was not designed to assess changes in oncology interest, knowledge, or awareness. Responses were subject to potential response and social-desirability bias, particularly given participants’ close interaction with program faculty. Because the program combined clinical exposure, mentorship, research participation, and financial support, the effects of individual program components cannot be separated. Detailed time-allocation data for individual clinical, didactic, laboratory, and research activities were not available retrospectively, limiting precise replication of the rotation schedule.

Prior work has demonstrated a lack of structured training in collaborative tasks between radiation oncology and medical physics trainees, with time constraints cited as a major barrier to interprofessional collaboration [9]. Additionally, bidirectional misunderstandings of professional roles can contribute to communication breakdowns, resulting in less efficient clinical workflows and fragmented patient care. One study noted that while other healthcare professionals often shadow physicians during training, physicians are less frequently exposed to the roles of other team members [10]. These observations are consistent with previously described challenges in multidisciplinary oncology education and warrant further study in larger cohorts.

Despite these limitations, participants reported positive educational experiences and subsequently engaged in oncology-related scholarly work. Future studies should include larger student samples, baseline and post-rotation assessments, comparison groups, and multiple institutions to evaluate educational and career-related outcomes associated with this approach. Additional study of opportunities for clinical and research involvement may also help inform the development of future oncology education programs.

## Conclusions

This pilot experience demonstrates the feasibility of providing early, structured multidisciplinary oncology exposure to preclinical medical students. Participants reported high satisfaction and subsequently engaged in radiation oncology scholarship. Although one participant later entered Internal Medicine and subsequently expressed interest in pursuing fellowship training in medical oncology, career interest was not formally assessed before and after the rotation; therefore, the effect of the program on specialty interest or career choice cannot be determined from this pilot experience.

## Appendices

**Table 1:** Post-rotation evaluation questionnaire.

Question	Response options
I felt that overall, the staff in my hosting department were knowledgeable and eager for my participation.	Very much so / Knowledgeable, but not eager / Eager, but not knowledgeable / Neither eager nor knowledgeable
Please rate your overall experience in your hosting department during your rotation.	Excellent / Very good / Good / Dissatisfied
Please evaluate the length of the Summer Rotation.	More time / About right / Less time
Please evaluate the amount of time spent in the Radiation Oncology Clinic.	More time / About right / Less time
For the time spent in the Radiation Oncology Clinic, please evaluate the amount of time spent shadowing the Radiation Oncology attendings.	More time / About right / Less time
For the time spent in Radiation Oncology Clinic, please evaluate the amount of time spent with the Radiation Therapists, Dosimetrists, or Physicists.	More time / About right / Less time
Please evaluate the amount of time spent shadowing other cancer physicians.	More time / About right / Less time
Please evaluate the amount of time spent in the Radiation Oncology Laboratory Space (observing bench research).	More time / About right / Less time
Free response questions:	
1. Please offer us advice from your perspective on how we can improve the experience for students at ROC.	
2. What was the most positive experience you had during your rotation at ROC?	
